# What is the effect of using collaborative assessment on symptomatology as an intervention in the treatment of mental illness? A systematic review and meta-analysis protocol

**DOI:** 10.1186/s13643-022-02124-x

**Published:** 2022-11-17

**Authors:** Oliver Rumle Hovmand, Ole Jakob Storebø, Nina Reinholt, Jasmin Rejaye Gryesten, Sidse M. Arnfred

**Affiliations:** 1 Region Zealand Mental Health Service, Research Unit for Psychotherapy and Psychopathology & Psychiatry South, Slagelse, Faelledvej 6, 4200 Slagelse, Denmark; 2 Department of Clinical Medicine, Faculty of Health, University of Copenhagen, Copenhagen, Denmark; 3 Region Zealand Mental Health Services, Psychiatric Research Unit, Center for Evidence-Based Psychiatry, Slagelse, Denmark; 4 Department of Psychology, Faculty of Health Science, University of Southern Denmark, Odense, Denmark

## Abstract

**Background:**

Clinicians usually conduct diagnostic assessments in order to establish a diagnosis or to evaluate the effect of treatment. Two meta-analyses suggest that diagnostic assessment administered in collaboration with the patient and personalized feedback might have a therapeutic effect.

**Methods:**

We aim to conduct a systematic review and meta-analysis of the effect on symptomatology when using assessment as a therapeutic intervention for patients with psychiatric illnesses.

We will search in five relevant electronic databases. Two reviewers will independently select papers following pre-defined eligibility criteria, extract data, and assess the quality of included studies. Randomized controlled trials comparing the effect of psychological assessment with other psychotherapeutic interventions in populations of patients will be included in the meta-analysis. We will extract data on symptom-related outcomes, quality of life, dropout, and re-diagnosis and use meta-analysis techniques to compute the effect size of interventions using assessment as a psychotherapeutic intervention.

The review will be conducted and reported according to the Preferred Reporting Items for Systematic Reviews and Meta-Analysis (PRISMA) statement. Risk of bias will be assessed by using the Risk of Bias tool RoB 2.0 of the Cochrane Collaboration, and the certainty of the body of evidence will be assessed with the Grading of Recommendations Assessment, Development and Evaluation (GRADE) approach.

**Discussion:**

**The results will be able to inform clinicians and policymakers on the effect of assessment and, depending on the results, could lead to a recommendation for modified assessment procedures and approaches in mental health services. Ultimately, it might improve the treatment outcome in mental health services.:**

**Systematic review registration:**

PROSPERO CRD42021270567

## Background

Psychological assessment has traditionally been viewed exclusively as a way to diagnose psychiatric disorders, gather information about patients’ and clients’ symptomatology, and plan and monitor treatment. This is described as the information-gathering model of assessment [1]. Recently, evidence has emerged which may suggest that psychological testing might be useful as a treatment if administered in a way that enlists the patient as a collaborator and not an “object” of the assessment. Several mental health professionals have developed frameworks for utilizing standardized psychological assessment as a therapeutic intervention, of which the major are the therapeutic assessment (TA) and collaborative assessment (CA) frameworks [2, 3]. For simplicity, will we from hereon describe these interventions with the acronym C/TA as utilized elsewhere in the literature [3].

C/TA refers to a range of semi-structured, brief, therapeutic interventions where a therapist (usually a psychologist) collaboratively administers standardized psychological tests and delivers feedback in a valuable and enriching way that can be therapeutic for the patient. C/TA therapists recognize that psychological assessment is an interpersonal event, and that the relationship between assessor and patient is paramount both concerning the validity of the result and in relation to the patient’s further treatment [3].

TA therapists emphasize respect for the patient, and the therapist recognizes that patients are “experts on themselves.” In the TA assessment, the therapist asks the patients what they wish to learn from the assessment at the beginning of the treatment. They do this by making the patient formulate a series of therapeutic questions they would like to “ask the tests” and work with the patients to find personally viable solutions to their problems and answers to these questions. This includes personalized feedback, both in oral and written form, based on results from each test administered and their answers to the patient’s therapeutic questions produced during the intervention. In addition, the patient is given a report of the assessment, formulated in simple language.

### *Proposed mechanism of action*

The existing literature on C/TA proposes the following key mechanisms of action of the interventions: (1) Finn and Tonsager (1992) suggested that the TA intervention combined aspects of self-enhancement and self-verification, and that the TA therapist during the intervention confirms aspects of the client’s self-schemata, which previously have not been verified by others [4]. This “self-verification” was in their research supported by findings from clients’ evaluations of the intervention. (2) Furthermore, in the TA intervention, the clients are exposed to different ways of thinking about themselves. When working through the psychological tests, the patients are offered objective information about why they might feel and act the way they do. As a result, patients may experience relief and can be offered strategies to alter behavioral patterns which cause them psychological distress [4]. (3) C/TA provides the clients with information on her psychological processes and psychopathology and therefore acts as specialized psychoeducation. Based on theories of self-psychology, Finn and Tonsager (1992) proposed that this “naming” and “explaining” of patients’ experiences which happen as a part of the extensive feedback in TA help strengthen patients’ “self-structure.” Furthermore, they hypothesized that this acted via strengthening and stabilization of the patient’s “self-structures,” which could prevent this from breaking down in times of severe stress [4]. (4) Finn and Tonsager (1997) further hypothesized that TA worked via “self-enhancement” based on theory from the object-relation school of psychotherapy. They noticed in their empirical research that many patients had negative self-images, which were reflected in their choice of therapeutic questions. During the TA intervention, the therapists try to change the patient’s narratives about themselves into one more positive, nuanced, and coherent [5]. (5) The patient develops a relationship with the C/TA accessor during the C/TA intervention, which will carry over to the therapeutic relationship with the psychotherapist handling her treatment. Hilsenroth, Peters, and Ackerman (2004) found empirical support for this in a sample of outpatients awaiting therapy and found that TA strengthens the therapist-client alliance, and that this alliance carried over to subsequent clinicians [6]. In relation to this, it is possible that the common factors in psychotherapy might contribute to the possible effectiveness of C/TA [7, 8]. (6) If clients lack progress in ongoing psychotherapy, C/TA aids the therapist in the reconceptualization of the case, and the new information helps her, and the patient moves forward in therapy.

### Why is it important to do this review?

The C/TA literature consists of textbooks [1–3], single-case studies, and a body of randomized controlled studies and noncontrolled studies. Many of these studies were included in a meta-analysis by Poston and Hanson (2010) focusing on C/TA in its broadest definition [9] and another meta-analysis by Durosini and Aschieri (2021) which focused on studies utilizing solely TA [10].

The meta-analysis conducted by Poston and Hanson (2010) included 17 randomized controlled or open clinical trials (*n* = 1496) on the effect of C/TA delivered in clinical psychology (*n* = 7) and counseling psychology (*n* = 10) settings [9]. They calculated an average effect size across all of the outcomes utilized in the included trials (*n* = 52) based on effect sizes extracted from the included studies and found a moderate effect of C/TA on outcomes between groups (Cohen’s *d* = 0.42 [95% *CI*, 0.321 to 0.525]). The authors concluded that “( … ) psychological assessment procedures - when combined with personalized, collaborative, and highly involving test feedback – have positive, clinically meaningful effects on treatment, especially regarding treatment processes” (p. 203) [9]. However, the study was limited by significant heterogeneity among the included trials regarding study design (inclusion of both randomized trials and non-randomized studies), subjects (ranging from healthy individuals receiving career counseling to individuals with suicidal tendencies), and outcomes (e.g., ranging from “weekly consumption of alcohol” to “session depth”). Furthermore, they did not place criteria on diagnostic assessment and had no definitions of clinical versus nonclinical sample. Neither did the study provide information on which studies used clinical and nonclinical samples. Furthermore, the review did not assess the risk of bias in studies or grade the certainty of the evidence as recommended for systematic reviews and meta-analyses [11]. Specifically, Lilienfeld, Garb, and Wood [12] criticized the review for having too wide inclusion criteria leading to overly wide sampling and results. Hanson and Poston (2011) [13] replied to this critique by publishing an updated version of their meta-analysis in which they excluded three studies and reanalyzed the remaining 14 studies (*n* = 1375). This updated version resulted in a statistically significant weighted Cohen’s *d* = 0.40 [95% *CI*, 0.30 to 0.50] across outcomes but added no improved reporting of populations, diagnostic criteria, or assessment of study quality or certainty of evidence.

Correspondently, Durosini and Aschieri (2021) focused in their meta-analysis on studies utilizing TA solely [10]. They reported on the results of nine primary studies (*n* = 491) comparing TA to a control group and calculated effect sizes for 42 different variables, grouped into three types of outcomes: treatment process (6 studies, 18 variables), clients’ symptoms (6 studies, 17 variables), and clients’ self-enhancement (5 studies, 7 variables). Furthermore, they conducted *Q* and *I*^2^ heterogeneity tests and moderator analysis. Their results revealed statistically significant intervention effects for each outcome between groups (treatment process: Hedges *g* = 0.46 [95% *CI*, 0.33 to 0.59]; *p* < .001, *Q* (17) = 8.62, *p* = 0.951, *I*^2^ = NA; clients’ symptoms: Hedges *g* = 0.34 [95% *CI*, .06 to 0.63]; *p* = .021, *Q* (16) = 19.26, *p* = 0.255, *I*^2^ = 32.94; clients’ self-enhancement: Hedges *g* = 0.37 [95% *CI*, .05 to 0.69]; *p* = .029, *Q* (6) = 7.110, *p* = 0.311, *I*^2^ = 19.37).

Based on these results, the authors advocated that implementing TA in clinical settings as an add-on intervention to other treatments could enhance treatment outcomes. However, the meta-analysis used heterogeneous samples (ranging from healthy individuals receiving career counseling to individuals with suicidal tendencies) with no criteria placed on diagnostic assessment and provided no information on which studies used clinical and nonclinical samples. Furthermore, the study used multiple outcomes and measures and mixed study designs (both randomized controlled trials and non-randomized studies), and did not assess the risk of bias in studies or grade the certainty of the evidence. In addition to this they did not utilize a proper technique to adjust *p*-values due to multiplicity [14]. Finally, the generalizability of results may be hampered due to the exclusion of C/TA interventions other than TA.

Thus, even following the updated review by Durosini and Aschieri (2021), do we still see the need for an updated systematic review and meta-analysis of C/TA. Especially, we find it important to conduct a systematic review and meta-analysis, which focuses solely on the use of C/TA in clinical populations. This is in order to quantify the effect of this family of interventions when used in for patients receiving treatment in a primary care setting or in mental health service outpatients or inpatients facilities.

The International Prospective Register of Systematic Reviews (PROSPERO; https://www.crd.york.ac.uk/PROSPERO/; accessed September 21, 2022) indicates one ongoing review concerning the use of assessment as an intervention in “Adults with mental health problems,” registered July 31, 2021 [15]. However, we foresee the following shortcomings of this ongoing review: (a) they propose to include both randomized clinical trials and non-randomized studies; (b) they do not propose to adhere to gold-standard Cochrane Collaboration methodological guidelines and do not plan to assess risk of bias with RoB 2.0 [16] and the certainty of the evidence [17]; or (c) adhere to the Preferred Reporting Items for Systematic Reviews and Meta-analysis statement (PRISMA) [18]. (d) Furthermore, the proposed review has unspecific inclusion criteria regarding the definition of what constitutes adults with mental health problems, and participants/populations are simply described as “Adults with mental health problems” (g) and plan to search only a limited number of resources.

Thus, we see the need for an updated systematic review and meta-analysis of C/TA, which investigates the efficacy of C/TA in clinical populations, employing sound research methods.

The following proposed review could quantify the effect of this family of interventions when used in clinical populations receiving treatment in a primary care setting or in mental health service outpatient or inpatient facilities. The results of such a study will be able to advise clinicians and policymakers in deciding which kind of assessment should be utilized and whether they should apply C/TA as a primary or a pre-therapy intervention. In addition, the review will provide a status of the evidence in the research field and provide data to guide the research field forward.

### Objective

To provide a synthesis of the effectiveness, benefits, and harms of collaborative diagnostic assessment as a treatment along with personalized feedback, compared to other interventions for adults in primary care or mental health service outpatient settings.

The review will follow the PRISMA guidelines [18], and this protocol will adhere to the PRISMA-P protocol guidance [19]. The review has been registered in the PROSPERO International Prospective Register of Systematic Reviews on August 28, 2021 (registration number: CRD42021270567).

## Methods/design

### Types of studies

We will include randomized controlled trials. This is defined as trials comparing two or more interventions in a population of patients who are randomly assigned to one of the interventions.

### Types of participants

We will include adult patients in primary care setting or secondary (mental health service) outpatient settings with all psychiatric diagnosis diagnosed according to both the American Psychiatric Association’s *Diagnostic and Statistical Manual of Mental Disorders* (DSM) III [20], IV [21], and V [22], and the WHO's International Statistical Classification of Diseases (ICD) 9 [23] or 10 [24], with or without comorbid conditions, except mental retardation.

### Types of interventions

Eligible trials will examine the effects of collaborative diagnostic assessment with standardized tests and instruments and personalized feedback as an intervention. Studies will complete this criterion if they describe utilizing the test as a part of the treatment and the tests are administered collaboratively with a therapeutic overlay. Tests can both when administered individually and in groups (as defined by the authors of the primary studies).

### Exclusion criteria

We will exclude studies on Collaborative Assessment of Suicidality (CAMS). We do so because we find the intervention better suited to be treated in a separate meta-analysis since we consider it a research field on its own different from C/TA.

### Types of comparator(s)

For inclusion in the meta-analysis, studies will require a comparator, which can be an assessment as usual or another psychotherapeutic intervention. Assessment as usual is defined as the administration of a psychiatric assessment without a therapeutic overlay and personalized feedback.

### Outcomes

Outcomes can be self-administered by patients or clinician administered. All outcome measures must be psychometrically validated.

#### Primary outcomes

##### Symptomatology

In the meta-analysis, we will include studies with a symptom-related outcome. If more than one symptom-related outcome is reported, we will use the one described as the primary outcome in the study. If no symptom-related outcome is described as primary, then NR, ORH, and JRG will make a consensus decision on the most appropriate outcome. The process will be documented in the final paper. We will use the reported end-of-treatment data for the primary analysis.

##### Adverse events

We will further report on outcomes on adverse events and use the European Medicines Agency definition of adverse events and serious adverse events [25]. We will (a) report on the risk ratio of experiencing any adverse events and (b) the risk ratio of experiencing a serious adverse event.

#### Secondary outcomes

##### Readiness for treatment

We will report on readiness for treatment measures. Due to the possibility of studies utilizing different measures for readiness for treatment, NR, ORH, and JRG will make a consensus decision of whether a measure is an appropriate measure for readiness for treatment.

##### Information sources

Five electronic databases are selected for searching: PubMed, PsycInfo, Web of sciences, Cochrane Central Register of Controlled Trials (CENTRAL), and Embase.

These sources will be searched using combinations of relevant search terms that we developed and tested for sensitivity in advance of the systematic review and meta-analysis, which will be reported in the appendix along with the dates that the searches were conducted.

Furthermore, we will supplement the literature search with a manual review of the reference lists from eligible publications, including textbooks on the subject.

We have included our full search strategy including mesh terms in the appendix to the present trial.

### Data collection and analysis

We will conduct this review according to guidelines set out in the *Cochrane Handbook for Systematic Reviews of Interventions* [26] and perform the analyses using the latest version of Review Manager 5 (RevMan 5), Cochrane's statistical software [27].

### Data management

The Covidence application [28] will be used to manage records throughout the review process [29]. The selection, removal, and rejection of studies will be documented in the PRISMA flow diagram [18].

### Selection of studies

After removing duplicate citations, studies will be screened in two stages: title/abstract and full text. Titles and abstracts of identified study reports will be independently screened by ORH and JRG using the inclusion and exclusion criteria described in the appendix. If they disagree, they will discuss the matter and receive a consensus. If they cannot reach an agreement, they will consult NR.

If the title and abstract are deemed relevant, the paper will be read in full by both ORH and JRG independently, again using the inclusion and exclusion criteria described in the appendix. If they are in disagreement, disagreements will be resolved through discussion. If an agreement cannot be reached, they will, again, consult NR. In case of the exclusion of a trial, reasons for doing so will be documented.

We will list relevant RCTs that do not fulfill the inclusion criteria with reasons for exclusion in the “Characteristics of excluded studies” tables. To ensure transparency of study selection, we will provide a flow chart per the PRISMA statement to show how many records have been excluded and for what reason and how many records have been included [19].

### Data extraction

#### Study and sample characteristics

In terms of general study characteristics, we will extract the following information:

- Author
- Publication year
- Location
- Study design (only RCT studies)
- Sample size
- Sample characteristics (age, biological gender, ethnicity, country, diagnosis)
- Setting (primary care or secondary care)
- Participation and attrition rates
- Intervention type (CA, TA, or other) and length
- Assessment instruments applied
- Outcomes relating to symptomatology
- Outcomes relating to readiness for psychotherapy
- Outcomes relating to adverse effects

### Data extraction and management

ORH and JRG will extract data, and ORH will enter the data into RevMan 5. They will resolve disagreements through discussion. In cases where there is not enough data or data is unclear in the published trial reports, we will contact the trial authors, requesting them to supply the missing information. We will develop data extraction forms to facilitate standardization of data extraction.

### Assessment of risk of bias in included studies

The risk of bias in the included RCTs in the meta-analysis will be assessed using the updated Risk of Bias tool RoB 2.0 of the Cochrane Collaboration [16].

For each included trial, ORH and JRG will independently evaluate each risk of bias domain as being at low risk, some concerns, or high risk, resolving disagreements by discussion. Each study will, following this initial rating, be rated overall according to its highest risk of bias in any of the assessed domains. However, we will rate studies with “some concerns” in multiple domains as “high risk” of bias [16]. Disagreement will be resolved via consensus discussion or consultation with NR.

We will assess the following domains in our risk of bias assessment: (1) bias arising from the randomization process, (2) bias due do deviations from the intended interventions, (3) bias due to missing outcome data, (4) bias due to measurement of the outcome, and (5) bias due to selective reporting [16].

#### Outcome data

To measure the effectiveness of the intervention in terms of our primary outcome, we will extract the following:

#### Continuous data

- Baseline symptom severity for all outcomes and if possible symptom severity at follow-up
- Duration of a possible follow-up measurement

#### Dichotomous data

- Amount of reported adverse events (yes/no) in percentage

## Measures of treatment effect

### Continuous data

We will compare the mean scores between the two groups to give a mean difference (MD) and present this with 95% CIs. We will use the overall MD to compare the outcome measures from trials. We will estimate the standardized MD (SMD), where different outcome measures are used to measure the same construct in the trials. We will calculate SMDs using both end scores and change scores (using standard deviations from the end score data) at post-treatment results and will also group the two different measures into independent groups and test for subgroup differences.

Our first choice will be to calculate effect sizes based on intention-to-treat (ITT) data. If means and standard deviations from an ITT analysis and missing values that were replaced are available, we will use these data. In other cases, we will conduct the analysis using only the available data.

We will report and utilize data on continuous outcomes reported at both ends of treatment and follow-up. We will utilize the reported number of patients, means, and SDs to calculate Cohen d’s effect sizes. We will interpret Cohen d’s effect sizes using the following general guidelines: small (0.2), medium (0.5), and large (0.8) [30].

In case of missing data, will we instead try to calculate these values from other reported values, such as *t*-tests and *p*-values.

If the outcome measure utilized in the studies measures a positive outcome with a high score compared to a low score, we will transform this value by multiplying −1.

### Dichotomous data

We will analyze the dichotomous data as risk ratios (RR) and present these with 95% CIs.

### Unit of analysis issues

We will analyze data at the end of treatment and the longest reported follow-up.

### Cluster-randomized trials

We do not expect cluster-randomized trials. However, if such trials are located in the literature search, we anticipate that investigators will have presented their results after appropriately controlling for clustering effects (robust standard errors or hierarchical linear models). If it is unclear whether a cluster-randomized trial has used appropriate controls for clustering, we will contact the investigators for further information. Where appropriate controls have not been used, we will request and reanalyze individual participant data using multilevel models that control for clustering.

### Studies with multiple treatment groups

If a trial compares more than two intervention groups, we will include all pairwise comparisons as long as they are not subject to the same meta-analysis.

### Dealing with missing data

We will try to obtain any missing data, including incomplete outcome data, by contacting trial authors. We will report this information in the “risk-of-bias” tables.

### Assessment of heterogeneity

We will group the heterogeneity of the reported trials into three domains: (1) clinical heterogeneity will refer to the possible variability in the patient populations regarding diagnoses, age, and treatment site. (2) Methodological heterogeneity will refer to differences regarding the intervention applied in the particular study. (3) Statistical heterogeneity is the difference in the intervention effects on the trials.

We will utilize *I*^2^ statistics for quantifying inconsistency in order to give an estimate on the percentage of variation in effect estimates, which can be regarded as due to heterogeneity rather than sampling error. We will judge *I*^2^ values of 0 to 40% as indicating little heterogeneity, 30 to 60% as indicating moderate heterogeneity, 50 to 90% as indicating substantial heterogeneity, and 75 to 100% as indicating considerable heterogeneity [26]. We will assess potential reasons for the heterogeneity by examining individual trial characteristics and subgroups.

### Data synthesis and analysis

We will perform statistical analyses according to recommendations in the latest version of the *Cochrane Handbook for Systematic Reviews of Interventions* [26]. In carrying out the meta-analysis, we will apply the inverse-variance method to give more weight to more precise estimates from studies with less variance (mostly larger studies). We will use the random-effects model for meta-analysis since we expect some degree of clinical heterogeneity to be present in most cases, though not so substantial as to prevent pooling in principle. We will prefer observer-rated measures as the primary analysis but will include self-reported outcomes if observer-rated outcomes are not available.

We will adjust the primary and secondary outcomes following the method described by Jacobsen et al. (2014) in order to adjust for multiplicity [14]. Multiplicity is about the concern that performing multiple comparisons increases the risk of falsely rejecting the null hypothesis [14].

### Sensitivity analysis

We plan to test any possible difference between “end-of-treatment scores” and “change scores.” We will conduct a sensitivity analysis to determine whether the findings are sensitive to random or fixed-effects models.

### Subgroup analysis

We will conduct the following subgroup analysis:

- Risk of bias (high risk of bias or low risk of bias accessed by the RoB tool)
- Biological gender (male and female)
- Length of the intervention (more or less than 10 h)
- Setting (primary care or secondary care)
- Intervention modality (TA, CA, or other)
- Age group (mean age < 30, 30–60, or > 60)
- Psychiatric comorbidity (yes or no)
- Referral diagnosis (emotional, psychotic, no diagnosis, or substance abuse)

### GRADE assessment

To assess the certainty of the body of evidence, we will apply the GRADE approach (Grading of Recommendations Assessment, Development and Evaluation) [17] as recommended by Cochrane [11]. We will use GRADE on our two primary outcomes: symptomatology and adverse effects.

## Discussion

Available evidence suggests that standardized psychological tests can be administered with a psychotherapy-oriented approach, which might benefit patients suffering from psychiatric illness [13]. The review described in the present protocol will provide an evidence synthesis of the effectiveness of administering standardized psychological tests as an integrative part of the treatment of patient populations with psychiatric illnesses.

There has already been one attempt to quantify the effects of psychological testing administered together with personalized feedback using meta-analysis techniques. However, these reviews were conducted with suboptimal techniques and had wide inclusion criteria.

In our review, we utilize contemporary standards, including assessment of bias, and focus solely on clinical populations. We will further exclude studies on Collaborative Assessment of Suicidality (CAMS) and do so because CAMS utilizes a test specifically developed for the purpose and does not utilize a standardized psychological test in a new way, as seen in the rest of the literature on C/TA. In addition, CAMS can be considered a research field in itself and is, therefore, better suited for treatment in a separate systematic review and meta-analysis.

We see some limitations to our design. C/TA is a heterogeneous field, and it is debatable which interventions should be regarded as C/TA and which should not. The heterogeneity of C/TA makes conducting a thorough literature search difficult. Thus, we might overlook psychological interventions that could be regarded as C/TA because they are not highlighted as being collaborative by the authors and therefore do not appear in the literature search. However, do we plan to conduct a manual review of the reference lists from eligible publications, including textbooks on the subject, which we believe will ensure literature saturation.

To the authors’ knowledge, this is the first systematic review and meta-analysis protocol that explicitly and exclusively intends to inform the development of therapeutic interventions that utilize assessment to treat psychiatric illness and their associated distress.

The review will provide an overview of psychological interventions utilizing psychological testing for purposes beyond diagnostic and treatment monitoring. The results might potentially improve the outcome of existing psychotherapeutic treatments, modify existing treatments, and be supportive when choosing treatment.

## Data Availability

The data used in the meta-analysis will be available from the corresponding author on reasonable request.
